# Ileal Gastrointestinal Stromal Tumor Presenting With Subacute Intestinal Obstruction and Synchronous Peritoneal Metastases: A Case Report

**DOI:** 10.7759/cureus.114770

**Published:** 2026-08-18

**Authors:** Benayad Aourarh, Omar Belkouchi, Lina Belkouchi, Samia Sassi

**Affiliations:** 1 Gastroenterology, Mohammed V Military Teaching Hospital, Rabat, MAR; 2 Department of General Surgery, International University of Rabat, Rabat, MAR; 3 Radiology, Faculty of Medicine and Pharmacy of Rabat, Mohammed V University in Rabat, Rabat, MAR; 4 Department of Radiology, Children's Hospital of Rabat, Rabat, MAR; 5 Department of Pathology, Mohammed V University in Rabat, Rabat, MAR

**Keywords:** gastrointestinal stromal tumor, ileum, imatinib, peritoneal metastases, small bowel obstruction

## Abstract

Gastrointestinal stromal tumors (GISTs) are rare mesenchymal neoplasms of the gastrointestinal tract. They most often arise in the stomach, followed by the small intestine. Small bowel GISTs usually present with gastrointestinal bleeding, anemia, abdominal pain, or nonspecific symptoms, whereas intestinal obstruction is uncommon because these tumors typically grow outward from the bowel wall. We report the case of a 58-year-old woman with no previous abdominal surgery who presented with one month of abdominal pain, constipation, and vomiting, which progressed to complete absence of stool and flatus for 48 hours. CT and MRI showed a 4.5-cm exophytic distal ileal mass with multiple peritoneal nodules and no liver lesions or lymphadenopathy. Because of the obstructive presentation, exploratory laparoscopy was performed.

Intraoperative findings showed a stenosing ileal tumor and diffuse peritoneal implants. Frozen-section analysis of two nodules suggested metastatic GIST, and segmental ileal resection with primary anastomosis was performed to relieve the obstruction and establish a definitive diagnosis. Histology showed a spindle-cell GIST positive for KIT (CD117) and DOG1, with a mitotic rate of 4/50 high-power fields. The peritoneal nodules were metastatic implants. The postoperative course was uneventful, and imatinib 400 mg daily was started one week later. After nearly two years of clinical and radiological follow-up, the patient remained stable without disease progression. This report highlights that ileal GIST should be considered in cases of unexplained small bowel obstruction, especially when imaging shows an exophytic mass without lymphadenopathy.

## Introduction

Gastrointestinal stromal tumors (GISTs) are the most common mesenchymal tumors of the digestive tract, although they account for less than 1% of all primary gastrointestinal neoplasms [1]. Their annual incidence is estimated at approximately 0.7-1 cases per 100,000 persons [2]. GISTs arise from the interstitial cells of Cajal and occur most often in the stomach, followed by the small intestine [3,4]. Small bowel GISTs may remain clinically silent for a long time because they usually grow in an exophytic pattern. When symptomatic, they commonly present with gastrointestinal bleeding, anemia, abdominal pain, or vague digestive symptoms. Intestinal obstruction is less common but may occur when the lesion causes luminal narrowing, acts as a lead point for intussusception, or, as in our case, when exophytic tumor growth promotes interbowel adhesions and bowel loop clumping [5,6].

Metastatic GIST most often involves the liver and peritoneum, whereas lymph node involvement is unusual [2,7]. The combination of subacute intestinal obstruction and synchronous peritoneal metastases at initial presentation is therefore uncommon and can mimic more frequent intra-abdominal malignancies. We report a case of ileal GIST presenting with bowel obstruction and peritoneal metastases, focusing on the diagnostic challenge, surgical decision-making, pathology, and clinical outcome during imatinib therapy.

## Case presentation

A 58-year-old woman with a history of asthma presented to our tertiary care center with a one-month history of abdominal pain, constipation, and vomiting. During the previous 48 hours, she developed a complete absence of stool and flatus. She denied fever, weight loss, night sweats, or gastrointestinal bleeding. She had no previous abdominal surgery, no known drug allergies, and no relevant family history of malignancy. She did not smoke or consume alcohol. On examination, the patient was alert, afebrile, and hemodynamically stable. Her BMI was 31 kg/m². Abdominal examination showed moderate distension with tympanic percussion and slightly hyperactive bowel sounds, without guarding, rebound tenderness, or other signs of peritonitis. Digital rectal examination was unremarkable. Laboratory tests revealed mild normocytic anemia, with a hemoglobin level of 11.3 g/dL. White blood cell count, renal function, liver tests, electrolytes, and inflammatory markers were within normal limits. Tumor markers were also normal, including a carcinoembryonic antigen level of 3 ng/mL and a CA 19-9 level below 2 U/mL (Table 1).

**Table 1:** Summary of the patient's laboratory findings at presentation

Laboratory test	Patient value	Reference range
Hemoglobin	11.3	13.0–17.0 g/dL
Mean corpuscular volume (MCV)	85	80–100 fL
White blood cell count	4,672	4.0–10.0 ×10⁹/L
Platelet count	223,000	150–400 ×10⁹/L
C-reactive protein (CRP)	4	< 5 mg/L
Serum creatinine	1.1	0.7–1.3 mg/dL
Blood urea nitrogen	9	7–20 mg/dL
Sodium	138	135–145 mmol/L
Potassium	4.0	3.5–5.0 mmol/L
Aspartate aminotransferase (AST)	11	10–40 U/L
Alanine aminotransferase (ALT)	15	7–56 U/L
Alkaline phosphatase (ALP)	65	40–130 U/L
Gamma-glutamyl transferase (GGT)	57	8–61 U/L
Total bilirubin	1	0.2–1.2 mg/dL
Albumin	4.1	3.5–5.0 g/dL
Carcinoembryonic antigen (CEA)	3	< 5 ng/mL
CA 19-9	2	< 37 U/mL

**Figure 1:**
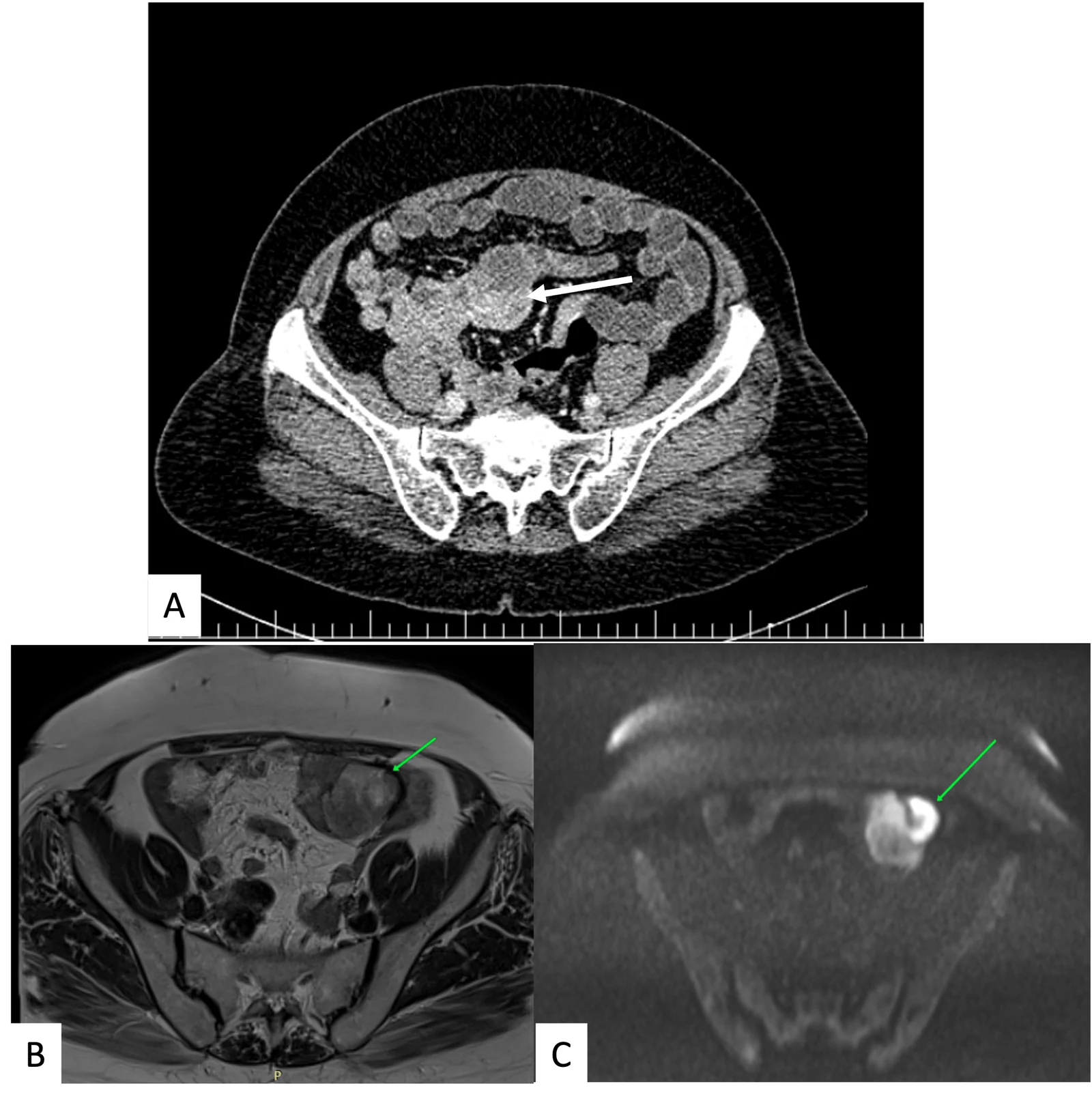
Imaging findings (A) Axial contrast-enhanced abdominal CT scan showing a 4.5-cm exophytic ileal mass with heterogeneous enhancement and a cystic component (white arrow), without lymphadenopathy or ascites. (B) Axial T2-weighted pelvic MRI demonstrating a well-defined ileal mass in the right iliac fossa with intermediate T2 signal intensity (green arrow). (C) Axial diffusion-weighted MRI showing high signal intensity of the lesion, suggesting restricted diffusion (green arrow), consistent with a gastrointestinal stromal tumor CT: computed tomography; MRI: magnetic resonance imaging

Contrast-enhanced CT demonstrated a 4.5-cm irregular exophytic mass arising from the distal ileum in the right iliac fossa, with heterogeneous enhancement and cystic areas. No intussusception, ascites, liver lesions, or enlarged mesenteric or retroperitoneal lymph nodes were identified (Figure 1). Pelvic MRI confirmed a solid-cystic terminal ileal lesion with intermediate T2 signal, low T1 signal, and restricted diffusion, findings suggestive of a hypercellular tumor (Figure 1).

Endoscopic evaluation was not attempted because of the risk of worsening the obstruction. The preoperative differential diagnosis included small bowel adenocarcinoma, lymphoma, neuroendocrine tumor, metastatic disease from colorectal or ovarian cancer, peritoneal tuberculosis, and GIST. The tumor’s exophytic relationship to the bowel wall, heterogeneous enhancement, cystic degeneration, absence of lymphadenopathy, and normal tumor markers favored a mesenchymal tumor, particularly GIST, although definitive diagnosis required tissue examination.

**Figure 2:**
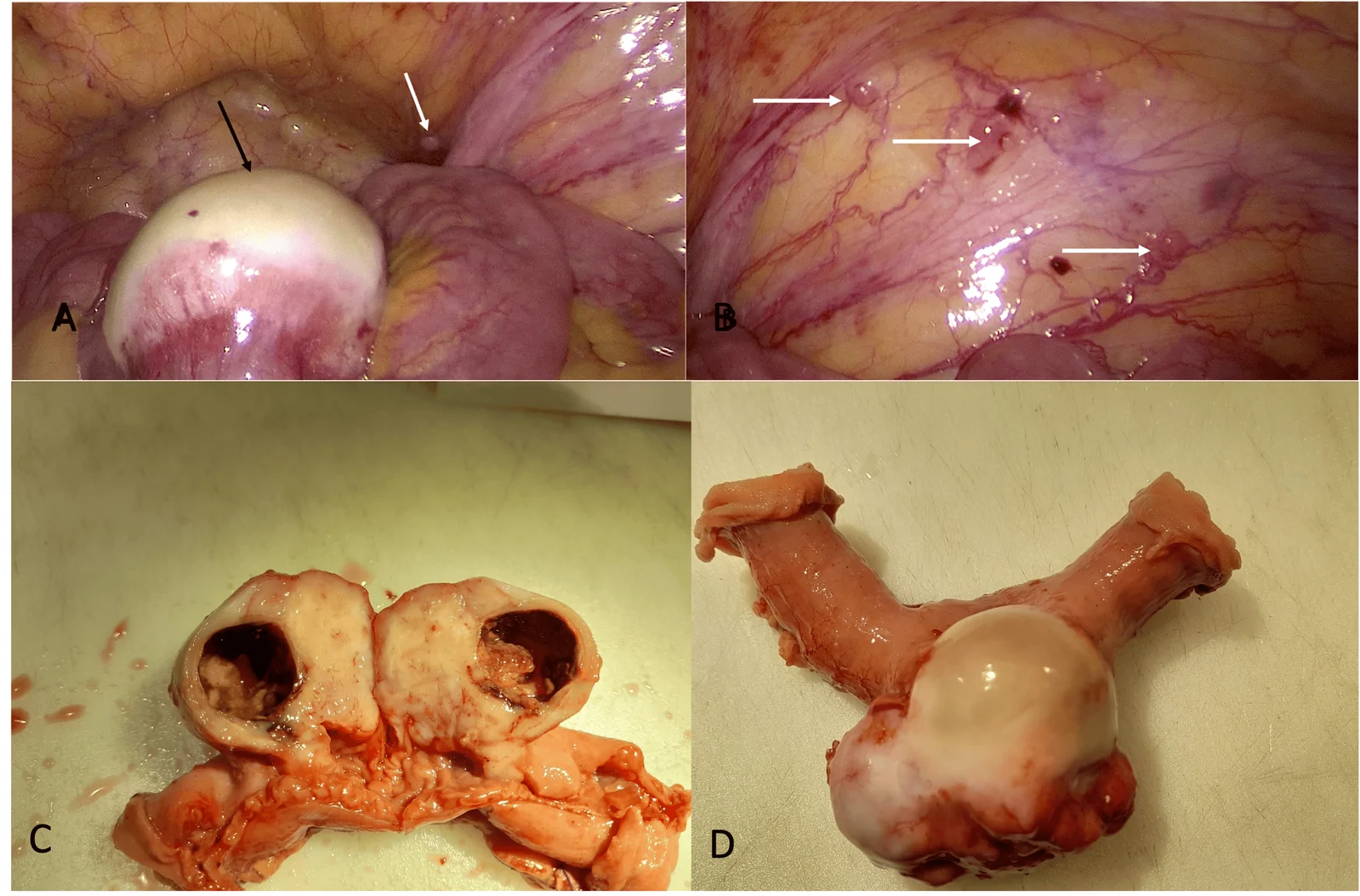
Laparoscopic and gross pathological findings (A) Laparoscopic view showing a stenosing ileal GIST with a smooth exophytic component (black arrow); the white arrow indicates a peritoneal metastatic implant. (B) Laparoscopic view showing multiple peritoneal metastatic implants on the peritoneal surface (white arrows). (C) External view of the resected ileal specimen showing a well-circumscribed exophytic tumor arising from the ileal wall. (D) Opened specimen demonstrating luminal narrowing with intratumoral necrotic/cystic and hemorrhagic areas GIST: gastrointestinal stromal tumor

Because the patient had progressive obstructive symptoms, the case was discussed in a multidisciplinary setting, and exploratory laparoscopy was chosen. The aim of surgery was diagnostic and symptom-relieving rather than elective cytoreduction. Preoperative management included bowel rest, intravenous hydration, and electrolyte correction. At laparoscopy, an exophytic stenosing tumor was identified on the antimesenteric border of the terminal ileum, approximately 15 cm proximal to the ileocecal valve. The proximal small bowel was moderately dilated. Multiple peritoneal nodules, ranging from a few millimeters to 1.5 cm, were distributed throughout the mesentery and parietal peritoneum (Figure 2).

**Figure 3:**
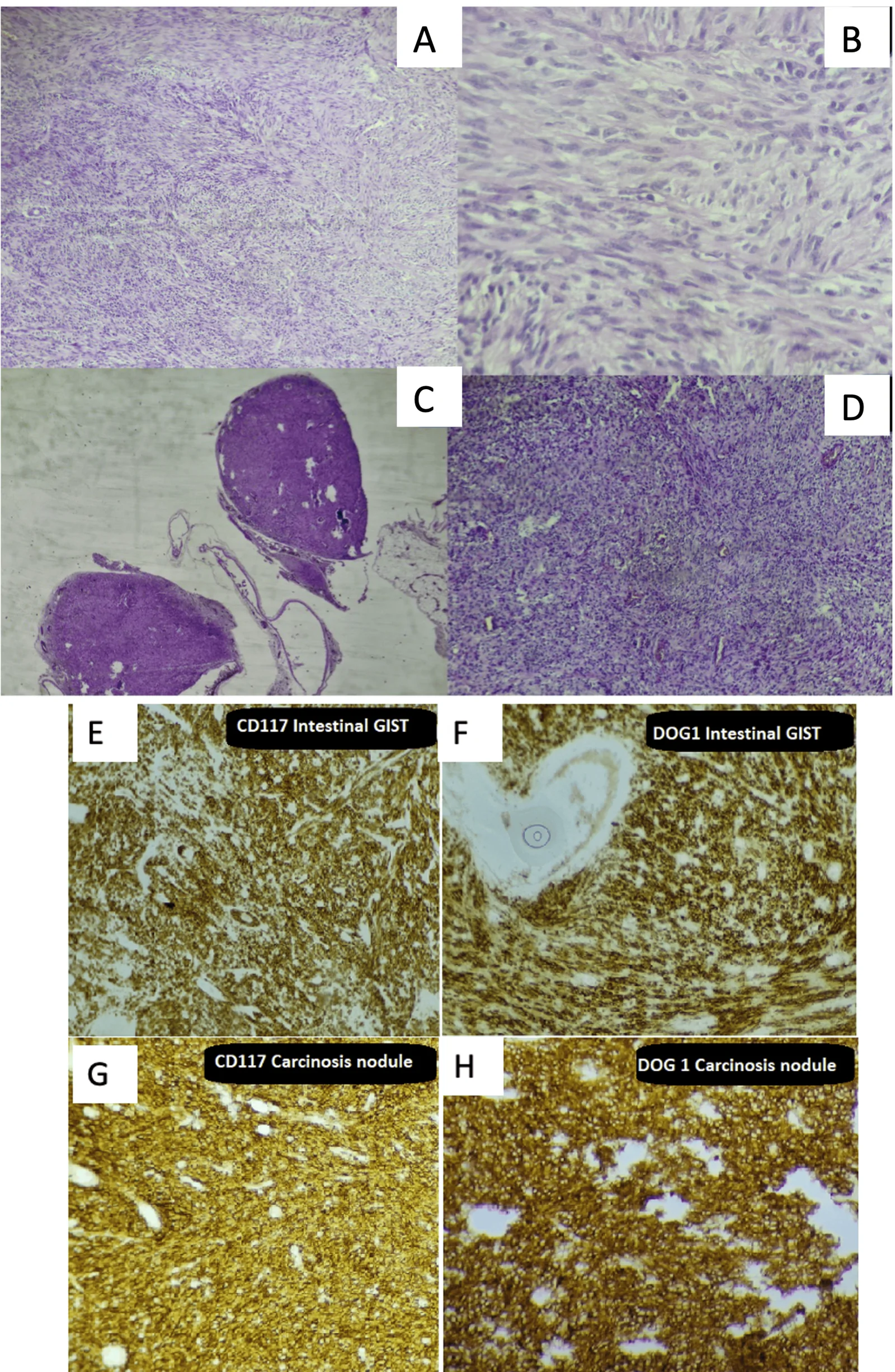
Histopathological and immunohistochemical findings (A, B) Hematoxylin and eosin staining of the primary ileal GIST demonstrating a spindle-cell neoplasm composed of elongated nuclei and eosinophilic cytoplasm with a hemangiopericytic vascular pattern. (C, D) Hematoxylin and eosin staining of a peritoneal metastatic nodule showing histological features similar to the primary tumor. (E, F) Diffuse immunohistochemical positivity for CD117 and DOG1 in the primary ileal GIST. (G, H) Diffuse CD117 and DOG1 positivity in the peritoneal metastatic nodule, confirming metastatic GIST GIST: gastrointestinal stromal tumor

Two accessible nodules were sampled from separate peritoneal sites and sent for frozen-section analysis. The result showed a malignant spindle-cell proliferation suggestive of metastatic GIST. Segmental ileal resection with primary end-to-end anastomosis was then performed to relieve the obstruction and obtain definitive pathology. Gross examination showed a well-circumscribed exophytic tumor measuring 5.2 × 4.0 × 4.0 cm with focal cystic degeneration. Microscopic examination showed a spindle-cell neoplasm composed of intersecting fascicles of elongated cells with eosinophilic cytoplasm and mild atypia. Mitotic activity was 4/50 high-power fields, and tumor necrosis was not identified. Immunohistochemistry showed diffuse positivity for KIT (CD117) and DOG1, focal positivity for CD34, and negativity for smooth muscle actin and S-100, confirming the diagnosis of GIST (Figure 3).

The sampled peritoneal nodules showed the same morphology and were consistent with metastatic implants. Formal microscopic margin status was not available in the pathology report. Molecular testing for KIT and PDGFRA mutations was not performed. In a localized setting, the tumor size, ileal location, and mitotic index would place the primary tumor in at least an intermediate-risk category according to commonly used risk stratification systems. However, the presence of synchronous peritoneal implants established metastatic disease at the time of diagnosis.

The postoperative course was uneventful, and the patient was discharged on postoperative day four. After discussion at the multidisciplinary tumor board, imatinib mesylate 400 mg daily was started one week after surgery. Follow-up included regular clinical assessment and interval cross-sectional imaging. After nearly two years of follow-up, she remained asymptomatic and clinically stable, with no radiological evidence of disease progression. She tolerated imatinib without clinically significant adverse effects and remained on the same treatment dose.

## Discussion

This case report is clinically relevant because it demonstrates how metastatic ileal GIST can present as a surgical problem and mimic more common causes of intra-abdominal malignancy. The main novelty lies in the convergence of four findings: subacute intestinal obstruction, synchronous peritoneal metastases without liver involvement, a preoperative appearance resembling peritoneal carcinomatosis from more common tumors, and durable stability following surgery and subsequent imatinib therapy.

The initial presentation raised several diagnostic possibilities. Small bowel adenocarcinoma can cause obstruction but is usually more infiltrative and may be associated with lymphadenopathy. Lymphoma can involve the small bowel but more often produces bulky wall thickening and nodal disease. Neuroendocrine tumors may produce a desmoplastic mesenteric reaction or liver metastases. Peritoneal tuberculosis was considered because peritoneal nodules may mimic malignancy, but the absence of fever, inflammatory syndrome, ascites, or systemic symptoms made it less likely. In this patient, the exophytic bowel-wall mass, cystic change, heterogeneous enhancement, and lack of nodal disease were more consistent with GIST [3,4].

Obstruction is uncommon in GIST because these tumors often grow away from the bowel lumen [5,6]. In the present case, the tumor was only moderately sized, but its location and stenosing relationship to the terminal ileum caused clinically significant narrowing. Similar obstructive presentations of ileal GIST have been described, but they remain rare [5,6]. Peritoneal metastases without liver lesions are also less common at presentation [2,7]. This is important because a low mitotic index does not exclude aggressive behavior, particularly in small bowel GISTs, which have a worse prognosis than gastric tumors of similar size and mitotic activity [4,8,9]. This apparent discrepancy between low mitotic activity and metastatic disease supports the need to interpret the mitotic rate together with tumor site, size, rupture status, and metastatic spread.

Surgery in metastatic GIST is individualized. In an asymptomatic patient with clearly metastatic disease, preoperative biopsy and first-line imatinib may be preferred. In our patient, direct surgical exploration was justified by the mechanical obstruction, the risk of complete obstruction, and the need for tissue diagnosis. The operation was not intended as complete cytoreduction; rather, it aimed to relieve the obstruction and confirm the diagnosis. Systemic treatment with imatinib remained the key oncologic therapy [4,10-12].

This case report has several limitations. Mutation testing for KIT and PDGFRA was not performed, although molecular profiling is important because it predicts response to tyrosine kinase inhibitors. Formal margin status was also not available in the pathology report. In addition, this is a single case, so the findings cannot be generalized. Despite these limitations, the case emphasizes a practical message: when small bowel obstruction is associated with an exophytic mass and peritoneal implants without lymphadenopathy, GIST should remain in the differential diagnosis, and surgery may still have an important role when obstruction is the predominant clinical issue.

## Conclusions

Ileal GIST is an uncommon cause of small bowel obstruction and may mimic more common intra-abdominal malignancies when peritoneal metastases are present. This report illustrates the diagnostic challenge posed by an exophytic ileal GIST presenting with subacute intestinal obstruction and synchronous peritoneal metastases without liver involvement. Prompt surgical intervention enabled definitive diagnosis, relief of bowel obstruction, and subsequent initiation of targeted therapy, resulting in sustained disease control during follow-up. Cross-sectional imaging may suggest the diagnosis when it demonstrates an exophytic small bowel mass with heterogeneous enhancement and no lymphadenopathy, but histopathology and immunohistochemistry remain essential for confirmation. In metastatic disease, surgery should not be considered routine cytoreduction; however, it plays an important role in relieving obstruction, preventing complications, and establishing a definitive diagnosis. Long-term postoperative imatinib therapy can provide durable disease stability in selected patients with metastatic GIST.
